# An Empirical Analysis of the Perceived Challenges and Benefits of Introducing Biosimilars in Bangladesh: A Paradigm Shift

**DOI:** 10.3390/biom8030089

**Published:** 2018-09-05

**Authors:** Eva Rahman Kabir, Shannon Sherwin Moreino, Mohammad Kawsar Sharif Siam

**Affiliations:** 1 Department of Pharmacy, BRAC University, 66 Mohakhali, Dhaka 1212, Bangladesh; sherwin.moreino@gmail.com (S.S.M.); sharif.siam@bracu.ac.bd (M.K.S.S.); 2 Darwin College, University of Cambridge, Cambridge CB3 9EU, UK

**Keywords:** biosimilars, Bangladesh pharmaceutical industry, biologics, interchangeability

## Abstract

The high demand for and resulting financial success of biopharmaceutical products over the last three decades have seen the door open for close copies of these biological products, also known as biosimilars. This paper seeks to collate all relevant published intelligence with acquired survey data to assess the weight of available evidence that these products hold immense potential for the pharmaceutical industry in terms of their applications and benefits. Biosimilars also pose to be of great promise to the Bangladesh pharmaceutical industry, with the commitment of drastically reducing its dependence on foreign imports of biopharmaceutics to meet local demand. Our questionnaire based survey involved 100 Clinicians, 50 Industry Experts and 100 Academicians. The study found that majority of Industry Experts (72%) and Academicians (63%) shared a different concept of biosimilars opposed to majority of Clinicians (78%). Majority of Academicians (68%) and Industry Experts (61%) also shared a different belief from that of most Clinicians (61%) regarding the need for updating the existing regulatory guidelines. The study also showed that Clinicians (67%), Industry Experts (83%) and Academicians (80%) highlighted the benefit of lower costs of biosimilars. Furthermore, the quality data obtained from the survey results allowed us to evaluate and provide recommendations for stakeholders on the need for increased biosimilar awareness, pharmacovigilance and safety in Bangladesh.

## 1. Introduction

The market for biologics (Appendix A1) and biosimilars is at a crossroads with uncertainties, as well as growing interest in their approvals and further development. The high costs and challenges of developing novel biotech products have led pharmaceutical companies to attempt at replicating existing products to maintain a steady stream of such biologics in the development pipeline [1,2]. These follow-on biological products, also known as biosimilars, are defined by the World Health Organization as products which are similar in terms of quality, safety and efficacy (Appendix A2) to an already licensed reference biotherapeutic product [3]. This new class of drugs aims to provide similar acute or chronic therapeutic response as their biological counterparts, without demonstrating a significant difference in efficacy, purity, potency (Appendix A3) and safety of administration [4]. Table A1 shows the timeline of biosimilars approved until 2018.

With the biotechnology industry moving towards more cost-effective ventures, facilitating biosimilar adoption poses to be of greater benefit to Bangladesh as opposed to the promotion of their reference (originator biologic) counterparts [5]. The sales of biosimilars such as Remicade have motivated manufacturers to promote more biosimilars in the drug development pipeline [6,7]. However, the road to desired biosimilar acceptance in such areas is replete with hindrances, especially those related to regulatory, manufacturing and commercializing concerns [8,9]. Overcoming such barriers requires substantial strategic planning, time and capital investment, as well as appropriate communication between stakeholders of the biosimilar industry. Table A2 identifies some current challenges to biosimilar introduction and their potential solutions.

Countries such as Bangladesh with a limited health budget, strong drug policies and a lower income earning population, strongly seek to benefit from the rapid growth of these products within the local industry [10]. Furthermore, an increase in demand for highly valued biologicals such as cardiovascular, antiasthmatic, anticancer and anti-diabetic medication has pushed pharmaceutical giants within South Asia to undertake a more holistic approach towards biosimilar development [11]. Internationally recognized regulatory bodies (such as the USFDA (US Food and Drug Administration), WHO (World Health Organization), EMA (European Medicines Agency) and IFPMA (International Federation of Pharmaceutical Manufacturers and Associations)) offer an abbreviated and streamlined approval process for biosimilars, which facilitates their commercialization if they can be shown to be highly similar to already approved reference products. This creates a potential opening for developing countries like Bangladesh into several international pharma markets such as that of the U.S and EU [12].

Biosimilars pose to provide alternate therapy options which may aid in promoting cost savings and competence to healthcare systems and subsequent ameliorated health outcomes in Bangladesh [13]. Table A3 illustrates the current landscape of biosimilars in disease treatment. Biosimilars possess unique characteristics with regard to small molecule generic drugs (Appendix A4) which pharmacists and Clinicians are required to understand in order to ensure these medications are used safely and optimally. The differences between small molecule drugs and biologics have been summarized in Table A4. A holistic understanding of the differentiation between small molecular, biological and biosimilar drugs have been described in Table A5. As a result of these differences, manufacturers of biosimilar products need to evaluate their immunogenicity (Appendix A5) [14] and interchangeability (Appendix A6) [15] prior to commercialization. Strong pharmacovigilance (Appendix A7) systems need to be proposed.

## 2. Case Study: Bangladesh Pharmaceutical Industry

Bangladesh holds a prominent position as a pharmaceutical manufacturer in South Asia, meeting both domestic and international demand. Similar to most other countries in the region such as Pakistan and India, it possesses well-structured regulatory pathways for the approval of pharmaceuticals [16]. Under the current national policy, raw materials locally manufactured are protected by restrictions on their import unless adequate quantity of the material is present within the local industry. Multinational companies (MNCs) are not allowed to market their products nationally without the setup of their own factories within the country. The marketing of foreign brands is also prohibited if there is the presence of at least three identical or similar products being locally produced [17]. The pharmaceutical industry in Bangladesh benefits from the Trade-Related Aspects of Intellectual Property Rights (TRIPS) waiver on pharmaceutical products for developing nations. However, this waiver is set to expire in 2033 [18]. In order to sustain growth after 2033, the industry must innovate and identify new opportunities. As of now, although the country meets most of its local pharmaceutical demand, it still relies on foreign imports for costly biotherapeutic products [19].

### 2.1. The Adoption of Pharmaceutical Biotechnology in the Pharmaceutical Industry of Bangladesh

With the patent expiration of most first-generation biologicals internationally in 2004, and new biologics having a patent (Appendix A8) period of just twenty years [20], prospects for developing biosimilars are brighter than ever. Taking advantage of the biosimilar movement would see Bangladesh keep up with other growing Southeast Asian pharmaceutical industries (such as those of India, Vietnam and Thailand) seeking the clinical and economic benefits of biosimilars [21]. However, there is currently a large gap in documented literature of the country’s biosimilar need, usage, regulatory policy and post marketing surveillance strategies which we aim to fill through this study. Although in its initial stages, the pharmaceutical industry of Bangladesh has steadily begun to employ biotechnology in the field of medicine. The industry aims to meet global pharma trends and reduce the local demand for biotechnology developed products. As a result, pharmaceutical companies are investing huge capital behind the development of anti-cancer, anti-HIV/AIDS, vaccines, insulin and several other biodrugs to meet local demand.

### 2.2. Concept of Non Comparable Biologics (NCBs) in Bangladesh

In Bangladesh, biosimilars tend to often be confused with non-comparable biotherapeutic products (also termed as biomimics [22]). These non-comparable biologics (NCBs) are copies of reference biologics which have not undergone the strict evaluations and regulatory requirements to meet biosimilarity [23]. The approval of these products is ambiguous since they lack data from comparative studies with the reference product. These products possess limited analytical evidence and clinical trial data, making it difficult to sufficiently compare their safety and efficacy profile with the licensed reference biologic. In certain countries with less stringent drug regulatory pathways, these NCBs are often marketed without clinical trials or sufficient disclosure of data to disclose their degree of biosimilarity, and are considered as biosimilars [24]. A clear perception of Non-Comparable Biologics is essential to distinguish them from biosimilars in terms of their safety and efficacy profile. Improving the understanding of the variations between the two would limit the number of low quality drugs reaching the Bangladesh drug market. Subsequently, this would also benefit patients undergoing therapy within the country.

### 2.3. Landscape of Current Regulatory Guidelines

Until the year 2017, the guidelines for biosimilar distribution and use were still being processed. The definition of biosimilars was set based on WHO biosimilar guidelines [3,25] as “a biotherapeutic product which is similar in terms of quality, safety and efficacy to an already licensed reference biotherapeutic product”. The approval process for bioproducts such as vaccines are currently handled by three expert committees. These three committees are the CMC (Chemistry, Manufacturing and Controls) committee, the clinical trial document evaluation body and the legal system utilized for all drugs within the policy. Approval is also directed by the Drug Control Committee and technical sub committees. However, under current guidelines, the Directorate General of Drug Administration (DGDA) promotes the operation and development of a pharmacovigilance system on biologics and other drug products. There are currently no regulatory boundaries set with regard to interchangeability issues of biologics and biosimilar drugs in the clinical setting. This means that the Clinician has to make an independent an informed decision on which drugs should be prescribed for treatment.

A guideline on the evaluation of biosimilar products was released by the DGDA at the start of the year 2018. This guideline is prepared in harmony with that of several globally accepted guidelines, such as the EMEA guideline on similar biological medicine, the WHO guideline on similar biotherapeutic products (SBPs) and the Korean guideline on Biosimilar products. This signals a large step forward for the country’s biosimilar industry in terms of attaining high standards with regard to global reputation. In order to obtain a better understanding of the presiding regulatory guidelines governing biosimilars within the country, data was collected from the reigning drug regulatory authority, the (DGDA [26].

## 3. Materials and Methods

After reviewing the general status of biologics and biosimilars globally, the current scenario in Bangladesh was researched in order to establish a case for the introduction and propagation of biosimilars within its drug industry. We built this case on a current view of the presence of biosimilars within the drug market. The information obtained was eventually related to the availability and accessibility of these drugs to the people of Bangladesh. The challenges involved in introducing, manufacturing and prescribing biosimilars within the present scenario were also highlighted.

### 3.1. Secondary Data

Secondary data for the study was compiled from several biologics and biosimilar related journals endorsed by Nature (London, United Kingdom), JAMA (Chicago, United States of America), Elsevier (Amsterdam, Netherlands), Springer (New York, United States of America) and other distinguished academia, relevant articles and guidelines. All the information collected were accurately referenced and compiled with the onus of providing a detailed understanding of biologics and biosimilars and their applications. Attempts were taken to identify any gaps or missing information within the literature.

### 3.2. Primary Data

#### 3.2.1. Identification of Stakeholders

For our analysis, it was necessary to first identify the major stakeholders with regard to biosimilar manufacture, prescription and education. We did this by answering the following three questions:

- Which stakeholders are most likely to recognize the specific approved industry standards required for biosimilar manufacture?
- Which stakeholders are most responsible for recognizing the value provided from a company’s biosimilar product in order to prescribe it?
- Which stakeholders hold the responsibility of promoting biosimilar awareness and education?

Table 1 below shows the stakeholders identified for the survey:

From the table we can see that Clinicians, Industry Experts and Academicians are the targets most desirable for the survey.

#### 3.2.2. Research Approval

The research was approved by BRAC University, 66 Mohakhali, Dhaka 1212, Bangladesh (approval code BRACU/PHR/2/2017/05). Furthermore, any information obtained that discloses the identity of any respondent has been excluded from the study.

#### 3.2.3. Design of the Questionnaire

Primary data for the study was obtained via the design and implementation of a questionnaire-based survey. Three sets of self-response questionnaires were made—each individually designed with questions targeting Clinicians, Industry Experts and Academicians respectively. The survey questionnaires were designed to address four specific objectives. These objectives are—(1) What is the status quo of the Bangladesh biosimilar drug industry? (2) How can the introduction of biosimilars benefit the health and welfare of the people of Bangladesh? (3) How can the adoption of biosimilars in the Bangladesh pharma industry be facilitated? (4) Are NCBs comparable to biosimilars? The questionnaires were tailored to answer these questions regarding the challenges and outcomes of biosimilar introduction and in demonstrating its feasibility in Bangladesh. The survey language was English and constituted of fifteen questions for Clinicians, twenty-five questions for Industry Experts and eleven questions for Academicians. The questionnaires constituted of multiple choice questions, Likert scales and open-ended questions aimed at allowing respondents to freely state their opinions on the discussion. Each question was prepared from output received from Focus Group Discussions (FGDs) involving established experts in the field of biosimilars. This was to ensure the Face Validity of the questionnaire. The three questionnaire sets were pre-tested on Clinicians (6), Industry Experts (10) and Academicians (6) to validate and develop the final questionnaire for each stakeholder. The full survey questionnaires used in this study is available as a supplemental material.

#### 3.2.4. Questionnaire Pretesting

During the pretesting process, it was found that pure probabilistic sampling methods alone could not be employed for the study since it would result in high non-response rates. Therefore, the selection for subjects within the targeted stakeholder groups were carried out utilizing Judgement and Snowball non-probability sampling methods. The selection criteria were dependent on the rational judgement of experts within the sample study who stood as representatives of the targeted stakeholder groups. Care was taken to ensure that the experts selected were adequately qualified to represent their stakeholder groups. The Snowball Sampling Method used is a non-probability sampling technique where the selection of additional respondents is based on referrals from the initial respondents. All respondents were notified by prior email and each respective administration was officially informed before the survey was conducted within the premises. Data collected during the survey was kept authentic by constant supervision from the survey taker. None of the respondents were allowed access to secondary information resources during the course of the survey. The questionnaires clearly mentioned that the survey would not require any of the respondents to provide their personal details. Respondents could refrain from answering any specific questions, or even choose to quit the survey at any point. The information from both primary and secondary sources were compiled and scrutinized before being incorporated into the study.

#### 3.2.5. Sample Size

We initially obtained the population size for each stakeholder group as follows:

(a) Clinicians: At the time of the study, the number of registered Clinicians was 85,890 [27]. This number was also validated during the Focus Group Discussions (FGDs). Questionnaires discarded during the screening process included those from Clinicians not currently prescribing biosimilars, and Clinicians who were unavailable during the survey period. Incomplete questionnaires due to confidentiality bindings and time limitations of the respondent were excluded during the post screening analysis. Also, questionnaires which did not provide quality information were excluded from the analysis.
(b) Industry Experts: For the study, we targeted only the top 30 pharmaceutical companies in Bangladesh. These are the companies which held 94.76% of the total market share [28]. We individually approached the Heads of each department within each company and were referred to the respondents, 157 of which were eligible for our study. This number was also validated during the Focus Group Discussions (FGDs). Questionnaires discarded during the screening process involved those returned from Industry Experts not currently involved in departments dealing with biosimilar manufacture and experts who were unavailable during the survey period. Incomplete questionnaires which did not provide quality information were excluded during the post screening analysis.
(c) Academicians: At the time of the study, we obtained the sample population size for Academicians, 26,319, from the University Grants Commission Annual Report [29]. This number was also validated during the Focus Group Discussions (FGDs). Questionnaires discarded during the screening process included those returned from Academicians who did not have biosimilars in their course content and Academicians who were unavailable during the survey period. Incomplete questionnaires which did not provide quality information were excluded during the post screening analysis.

We then computed our sample size from the population size for each individual stakeholder group using Daniel’s Equation [30] employed in Raosoft Inc.’s Sample Size Calculator [31]. For each sample size calculation, we adequately fixed a margin of error of 5%, a confidence level of 95% and a response distribution of 50%. The formula is as follows:

$$n=\frac{Z^{2}P\left(1-P\right)}{d^{2}}$$

where *n* = sample size, *Z* = Z statistic for a level of confidence, *P* = expected prevalence or proportion and *d* = precision.

The respondents were then approached to fill out the questionnaires. Questionnaires were screened in order to obtain only those which provided data relevant to the study. This included excluding questionnaires where the respondent was not involved in biosimilar manufacture or prescription or those where the respondent expressed no interest in being involved in the survey. After the screening process, we checked and discarded any questionnaire that was left incomplete by the survey taker. Final sample size for the study was tabulated.

Questionnaires filled in from the survey were then screened based on individually preset criteria and degree of completion. For Clinicians, the sampling frame included doctors practicing in hospitals within Dhaka city, who are well versed on the applications and benefits of biosimilars and biologics, as referenced by the director of each hospital. The sampling frame for Industry Experts included only those from companies either currently manufacturing or are planning to manufacture or market biosimilar products in the near future. Departments in these companies dealing with biosimilar products were only approached. We discarded any data obtained from Industry Experts whose companies were not involved in biosimilar production and/or marketing. Academicians who included biosimilars in their course content were targeted for the survey, based on references or counsel provided by the Dean of the respective departments.

**Figure 1:**
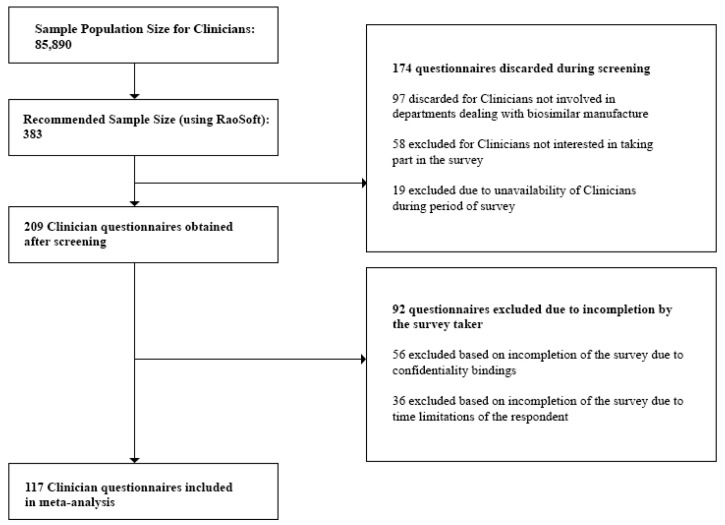
Questionnaire screening process for Clinicians.

**Figure 2:**
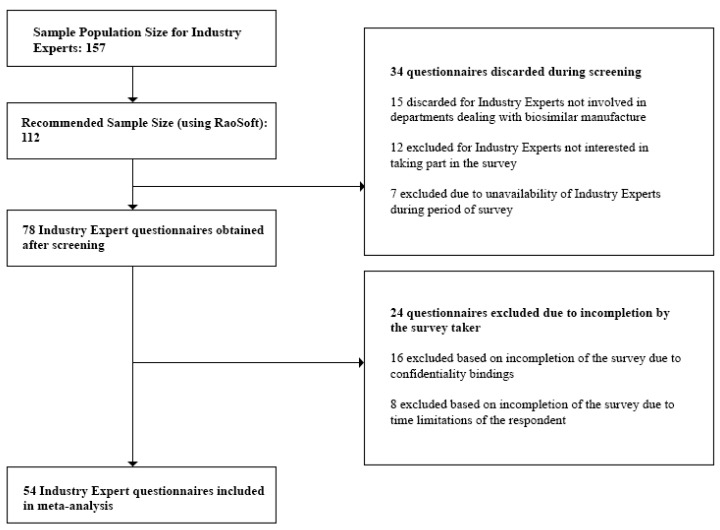
Questionnaire screening process for Industry Experts.

**Figure 3:**
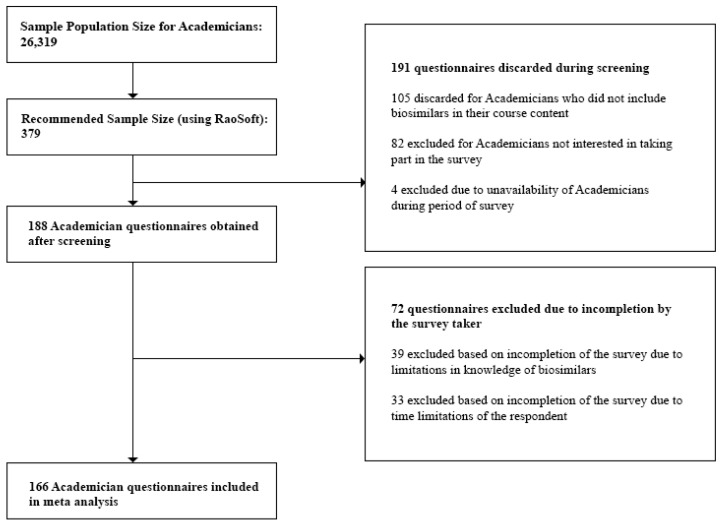
Questionnaire screening process for Academicians.

Figure 1, Figure 2 and Figure 3 below show the questionnaire screening process for each stakeholder group as well as the final number of questionnaires included in the meta analyses.

The minimum sample size or required number of subjects surveyed to represent the individual stakeholder groups was finally found to be:

(i) Clinicians—117
(ii) Industry Experts—54
(iii) Academicians—116

Lastly, we rejected any questionnaire which did not provide quality interpretable data and concluded with the following sample sizes to represent the individual stakeholder groups:

(i) Clinicians—100
(ii) Industry Experts—50
(iii) Academicians—100

Total number of responders (*N*)—250

### 3.3. Purpose of the Study

In light of the purpose of the study, we examined whether biosimilars introduced in the Bangladesh drug industry required any further clarification with regard to its industrial manufacture, distribution and clinical prescription. For this, we first drew an analogy between the concept of biosimilars among Clinicians, Industry Experts and Academicians, followed by contrasting the perceived challenges and benefits of biosimilars by the three stakeholders.

## 4. Results

The answers obtained from the respondents of the survey were analysed and interpreted, to relate to the leading research proposal. Discrepancies in data due to a lack of coherence and correlation among the survey answers were also identified. For some questions in the survey, feedback was not obtained from all three stakeholder groups due to confidentiality bindings and lack of quality interpretable data from those surveyed. Complete survey data was obtained from 50 Clinicians, 40 Industry Experts and 50 Clinicians within the three target sampling frames. Certain questions were also preformulated during the focus group discussions and pilot testing of the questionnaire to be targeted towards particular stakeholder groups of the survey. Information obtained from these questions was not utilized during the comparative analysis between stakeholder groups, but rather to fill out gaps in literature.

### Survey Feedback

We surveyed Clinicians, Industry Experts and Academicians within our sampling frame on their understanding of biosimilars, asking them to select a definition that reflects their concept of biosimilars. Answers obtained are presented in Table 2.

**Figure 4:**
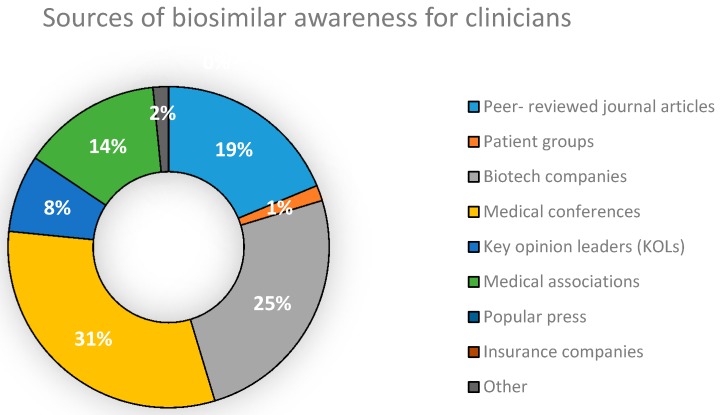
Sources of biosimilar awareness for Clinicians in Bangladesh.

In addition, we surveyed Clinicians within the sampling frame on the main sources from which they had been made aware of the existence, applications and benefits of biosimilars. Their feedback was compiled and presented in Figure 4.

**Figure 5:**
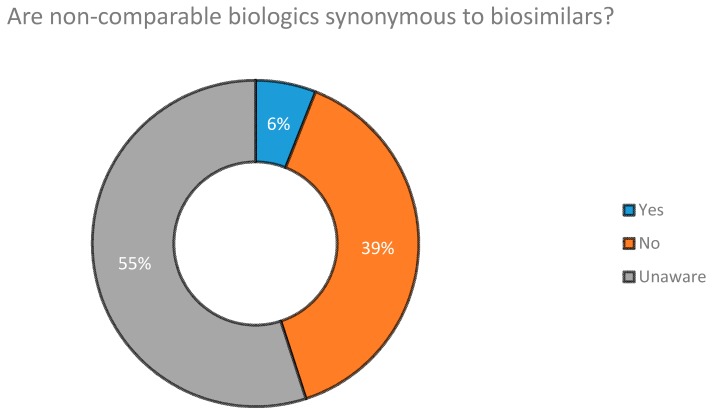
Are NCBs synonymous to biosimilar drugs according to Industry Experts.

When we surveyed Industry Experts on whether NCBs are synonymous to biosimilars, we found that a majority of those surveyed (55%) stated that they were unable to distinguish between the former and latter. Their answers are presented in Figure 5.

Clinicians, Industry Experts and Academicians were surveyed on the major challenges they associated with the introduction of biosimilars in the Bangladesh pharmaceutical industry. Their answers are compared in Table 3.

Additionally, Industry Experts were surveyed on what they perceived were the drivers which would motivate Clinicians to utilize biosimilars in their practice. Majority (61%) of their opinions favoured safety of the biosimilar product as being the primary driver of biosimilar prescription. Their responses are given in Table 4 below.

**Figure 6:**
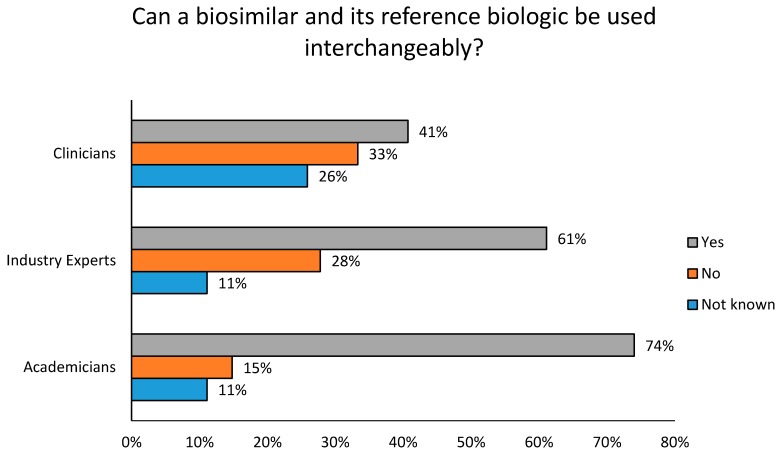
Are biosimilar products interchangeable with their reference biologics?

As previously mentioned, interchangeability of the biosimilar product with its reference biologic would significantly improve the adoption of biosimilars within the national drug industry. Industry Experts, Clinicians and Academicians were surveyed on whether biologics and their corresponding biosimilars could be freely utilized in place of each other during drug therapy. Their feedback is presented in Figure 6.

We surveyed Industry Experts regarding how biosimilar manufacturers can provide evidence to Clinicians regarding the reliability of their product, especially in terms of non-immunogenicity. 44% of Industry Experts felt that the provision of Phase III clinical trial data from a sample of the local population would strongly increase the assurance to Clinicians in prescribing the drug. Their feedback is given in Table 5.

Furthermore, Industry Experts were also surveyed on how patient awareness of biosimilar drug products can be strategically increased. Majority of their opinions (67%) favoured utilizing media for public counselling of patients. Their feedback is shown in Table 6.

With regard to the welfare of the people in Bangladesh and the rest of the world, Clinicians, Industry Experts and Academicians were surveyed to obtain their opinions on the advantages of biosimilar medication. Their feedback was compiled and presented in Table 7.

## 5. Discussion

While our study integrated information on biosimilars from several reputed journals, we observed a lack of documented and accessible data corresponding to the Bangladesh biosimilar industry. Our study was designed to identify any possible gaps in current literature that could pose as a barrier to desired biosimilar acceptance within the country, through the evaluation of the complications and rewards of biosimilar prescription in the status quo. Therefore, the results we obtained are broadly discussed in this section under three categories—Concept and awareness, Challenges in biosimilar introduction and Benefits of biosimilar therapy in Bangladesh.

### 5.1. Concept and Awareness

With regard to the concept of biosimilars, a majority of Industry Experts (72%) and Academicians (54%) regard biosimilars as drugs possessing equivalent clinical properties as that of the reference drug molecules. Their stance mandates the need for biosimilars to display identical safety and efficacy profiles compared to their reference biologics after undergoing Phase I-III trials. However, majority of Clinicians (41%) viewed biosimilars as those which demonstrate bioequivalence with the original biodrug and do not require clinical trials to be approved (Table 2).

Medical conferences were found to be the biggest source of awareness of biosimilar drug products for Clinicians practicing in Dhaka. The second biggest source was found to be companies working with biotech products (Figure 4). With regard to whether NCBs are synonymous and can be substituted with biosimilars, results obtained (Figure 5) show that 39% of Industry Experts surveyed disagreed that they were identical to each other. However, the concept of NCBs was still vague among a large proportion of industry professionals (55%), a matter which could potentially decrease the quality of drug therapy within the country. This increases the importance of the need to raise awareness of NCBs among Industry Experts, Clinicians, Academicians and patients. Doing so would sufficiently preserve the integrity of future biodrug treatment programs.

### 5.2. Challenges in Biosimilar Introduction

When analyzing the challenges involved in introducing biosimilars within the drug industry, the results obtained (Table 3) show that majority of Industry Experts (61%) and Academicians (68%) polarized towards the need for updated regulatory guidelines to facilitate the introduction of biosimilars into the Bangladesh drug industry. Majority of Clinicians (61%), however, viewed the overreliance of clinical data extrapolation to measure biosimilar effectiveness to be the greatest challenge needed to be overcome to establish effective biosimilar therapy. At the same time, there was also a high demand from Industry Experts and Clinicians for improved pharmacovigilance catering to biosimilar drugs while Academicians stipulated the need to reduce any patient health related complications which may occur during substitution of biosimilars in therapy. All three groups felt that the economic and societal consequences of biosimilar use would be insignificant if they were introduced after sufficient clinical testing and were regulated by standard guidelines. The demand from Industry Experts for an improved biosimilar postmarketing surveillance system to ensure drug safety is also reflected in their opinion of drug safety. Ensuring the safety of biosimilar products was the biggest driver of Clinician prescription of biosimilar products (Table 4).

The study suggests that Academicians (74%), Clinicians (41%) and Industry Experts (61%) felt that biosimilar therapy was interchangeable with their innovative drug therapies (Figure 6). They also supported its promotion within the clinical setting. A comparatively higher percentage of Clinicians (26%) within the city were doubtful regarding the interchangeability of therapy and 33% of Clinicians were against drug substitution, feeling that there were significant patient risks involved. Manufacturers who provide Phase III clinical trial data, preferably sampled in the local population, aided in bridging the gap of Clinician biosimilar awareness (Table 5). This also makes it relatively easier for Clinicians to prescribe their product in therapy. With regard to patient awareness, Industry Experts primarily recommended the utilization of public counseling campaigns through various media outlets for effective patient guidance. Patient counseling programs funded by pharmaceutical manufacturers, hospital boards or Clinicians (50%) was also well supported. However, a minority of Industry Experts (17%) supported patient education provided by hospital personnel during drug prescription. They considered this to be time consuming and disadvantageous to patients who needed medication in a state of emergency (Table 6).

### 5.3. Benefits of Biosimilar Therapy in Bangladesh

One of the advantages of biosimilars is its promise of potentially lowering healthcare costs (voiced by 83% of Industry Experts, 80% of Academicians, and 67% of Clinicians). It also assures to treat the same indications as those remedied by the reference biologic (voiced by 44% of Industry Experts, 44% of Clinicians and 35% of Academicians) (Table 7). The possible advantages of a lower therapeutic dose or utilization of an administration route varying from the original biologic were less favoured by the candidates surveyed.

## 6. Recommendations

Several countries remain skeptical to the introduction of biosimilars and the positive impact they promise to have on healthcare costs and bio-therapeutical outcomes. It is thus vital for concerned authorities around the world, including Bangladesh, to bridge the gap between biosimilar science and patients within the medical community. It is important to remove the concerns present regarding the use of follow on biologics. The current study identifies and recommends the need for multiple stakeholders i.e., pharmaceutical companies, regulatory authorities, Clinicians, Academicians to take a strong initiative in ensuring the benefits of modern medical technology. Furthermore, this information also needs to reach patients in a comprehensible manner. This would provide solutions to any critical issues and challenges that hinder the adoption of biosimilars in markets all over the world. Clear, authentic and easily accessible information regarding the concept of biosimilars should be given. We also recommend providing information of their approval process, their differences from brand name drugs and their accurate positions in drug substitution and interchangeability. We believe this will significantly improve Clinician and patient acceptability of biosimilar treatment options. Simultaneously, greater regulatory clarity and advocacy of a sustainable biosimilar market with forecasted cost savings and healthy competition would incentivize manufacturers to move towards greater biosimilar production. Lower pricing of biosimilars could benefit the patients, as well as present an opportunity to gain market share.

As seen from the results, it would be recommendable to reduce any areas of variation in the understanding of biosimilars among Clinicians, Industry Experts and Academicians. Medical associations and peer reviewed journal articles should be utilized to a greater extent in communicating new biosimilar developments to Clinicians. Guidelines issued should allow for clear concise differentiation between biosimilars and non-comparable biologics in order to facilitate drug prescription. The results infer the strong relation between effective pharmacovigilance systems and biosimilar commercialization. The study also shows a rise in demand for the promotion of the transparency of safety and efficacy data of biosimilar drugs to both Clinicians and patients. We also recommend that biosimilars only get approved after undergoing satisfactory Phase III trials. Since safety is the major motivator for clinical drug prescription, it is vital for biosimilar manufacturers to maintain pristine standards of drug manufacturing practices during the production process. Provision of evidence that WHO guidelines have been maintained, a move that is not upheld by national manufacturers, is also recommended during product registration to meet international standards. With only a few biosimilar drugs currently on the market, strategic methods need to be designed in order to properly access the marketplace, the achievement of which would see the country reach new heights in catering to its long-term healthcare needs. The greatest benefit of biosimilars anticipated by the respondents is its lower cost as opposed to their originators. Therefore, we recommend manufacturers to consider keeping a reasonable price difference between the two products in order to ensure market success.

The results that have been obtained from the survey may be used for future review of the existing national policy framework. The results may also be utilized to identify any areas of congruency the national drug policy may have with internationally recognized and accepted policy systems (such as the guidelines provided by WHO, EMA and USFDA). The results can be used to mark areas for development in the biologic and biosimilar drug industry. The feedback obtained from the study may aid in moulding prospective policies with a greater focus on the health and welfare of the people of Bangladesh.

### Limitations

There was a difference between the computed sample size and final number of respondents, the reasons being:

(a) only respondents who were experts in the targeted field of study were approached
(b) certain respondents were unable to provide feedback due to confidentiality bindings
(c) respondents were unavailable during the survey period

As a result, the statistical power was low. Thus, we did not report the inferential statistics.

## 7. Conclusions

In the past decade, biologics and biosimilars have definitely gained significant traction in the pharmaceutical market [32]. It is imperative that regulatory professionals worldwide, including those in Bangladesh, are equipped with the necessary knowledge and skills to evaluate the quality, safety and efficacy of such products.

Bangladesh continues to be one of the strongest frontier countries in the growth of pharmerging markets in the Asia Pacific [33,34], with over 3600 pharmaceutical brands of Bangladesh internationally registered. The country’s staggering population has not hindered its economic growth stability, nor its increasing health awareness through the successful development and propagation of health education programs and NGOs. There is currently a steadily growing awareness of biosimilars within industries, hospitals and universities of Dhaka, backed by its potential as a more prevalent option for future biodrug therapies. The biosimilar regulatory guideline recently published by the DGDA outlines the different criteria for biosimilar comparability testing, prescribing information and labelling. However, there are some limitations with regard to information on the manufacturing parameters, presence of clinical data, interchangeability and pharmacovigilance. All respondents agreed that the introduction of biosimilars might bring numerous benefits to the welfare of the people of Bangladesh. These would be mainly in the form of improving the accessibility of medication to patients, as well as offering a greater range of treatment options to Clinicians. This will further assist Clinicians in their decision making. It would be important to ensure that this does not intervene on a patient’s right to choose from a wider range of treatment options. This would also significantly boost patient acceptance, and Clinicians’ confidence in prescribing biosimilars. Tackling the challenges of biosimilar commercialization would encourage more pharmaceutical companies to join the global biosimilars market. As the Bangladesh pharmaceutical industry moves forward, companies operating in the country need to remain financially viable and competitive in terms of global quality standards. With meticulous planning, significant financial investment and enhanced awareness, the industry holds immense potential in positioning itself in the biosimilar market.

## Figures and Tables

**Table 1:** Identification of stakeholders to be targeted for the survey.

Stakeholders in the Value Chain of the Pharmaceutical Industry	Which Stakeholders are Most Likely to Recognize the Specific Approved Industry Standards Required for Biosimilar Manufacture?	Which Stakeholders Are Most Responsible for Recognizing the Value Provided from a Company’s Biosimilar Product in Order to Prescribe it?	Which Stakeholders Hold the Responsibility of Promoting Biosimilar Awareness and Education?
Clinicians	-	X	X
Patients	-	-	-
Industry Experts	X	-	X
Academicians	-	-	X

**Table 2:** Understanding of biosimilars among Industry Experts, Academicians and Clinicians.

*A Biosimilar is…..*	Industry Experts (N_IE_ = 20%)	Academicians (N_A_ = 40%)	Clinicians (N_C_ = 40%)
a biologic that demonstrates equivalence with the original biodrug and has all the preclinical and clinical trials equal to those already performed with the original biodrug	36 (72%)	63 (63%)	22 (22%)
a biologic that demonstrates bioequivalence with an original biodrug and does not need clinical trials to be commercialized	14 (28%)	37 (37%)	78 (78%)

**Table 3:** Challenges of biosimilars according to Industry Experts, Academicians and Clinicians.

	Industry Experts (N_IE_ = 20%)	Academicians (N_A_ = 40%)	Clinicians (N_C_ = 40%)
Need for updated regulatory guidelines	30.5 (61%)	68 (68%)	17%
Need for improved pharmacovigilance systems to ensure drug safety	28 (56%)	41 (41%)	56 (56%)
Extrapolation of clinical data may be insufficient to determine effectiveness of biosimilar	25 (50%)	10 (10%)	61 (61%)
Complications during substitution of drug in patient therapy	11 (22%)	56 (56%)	44 (44%)
Economic and societal consequences of biosimilar use	5.5 (11%)	0 (0%)	0 (0%)

**Table 4:** Drivers for Clinicians to prescribe biosimilars (According to Industry Experts).

Drivers for Clinicians to Prescribe Biosimilars	Response of Industry Experts (%) (N_IE_ = 100%)
Lower price of the biosimilar in comparison with the innovator biodrug	28 (56%)
Country of origin where the biosimilar has been manufactured	8.5 (17%)
Certified approval for the biosimilar by the relevant authorities	22 (44%)
Good manufacturing practices and high reputation of the manufacturer	19.5 (39%)
Bioefficacy of the biosimilar drug	22 (44%)
Safety of the biosimilar drug	30.5 (61%)

**Table 5:** Methods of biosimilar reliability demonstration by Industry Experts.

Practice for Demonstrating Reliability of the Product by Manufacturer	Response of Industry Experts (%) (N_IE_ = 100%)
Provision of bioequivalent safety and efficacy data	14 (28%)
Provision of data of Phase III clinical trial outcomes within a sample of the local population	22 (44%)
Provision of evidence of strong GMP maintenance	8.5 (17%)
Provision of evidence that WHO guidelines have been followed during product registration	5.5 (11%)

**Table 6:** Patient Education strategies on the use of biosimilars.

Patient Education Strategy	Response of Industry Experts (%) (N_IE_ = 100%)
Public counselling campaigns through various appropriate media	33.5 (67%)
Patient counselling programs sponsored by pharmaceutical companies and hospitals	25 (50%)
Patient education provided by hospital personnel during drug prescription	8.5 (17%)
Patient education through open seminars or symposiums held by Academicians from universities citywide	14 (28%)

**Table 7:** Benefits of biosimilars according to Academicians, Industry Experts and Clinicians.

Benefits of Biosimilar Medication	Academicians (N_A_ = 40%)	Industry Experts (N_IE_ = 20%)	Clinicians (N_C_ = 40%)
Lower cost	80 (80%)	41.5 (83%)	67 (67%)
Commercialization approved with initial indication including all diseases previously approved for the innovator biodrug	35 (35%)	22 (44%)	44 (44%)
Administration route different from that of the original biodrug	14 (14%)	3 (6%)	0 (0%)
Lower therapeutic dose	7 (7%)	0 (0%)	0 (0%)

## Supplementary Materials

The following are available online at http://www.mdpi.com/2218-273X/8/3/89/s1. Supplementary file 1: full survey questionnaires used in this study.

## Appendix A. (Terminologies)

A1: Biologics—Drugs that are made from living organisms or their products and used in the prevention, diagnosis, or treatment of cancer and other diseases. Biological drugs include antibodies, interleukins, and vaccines.

A2: Efficacy—Efficacy is a drug’s capacity to produce an effect (such as lowering blood pressure). e.g., diuretic furosemide eliminates much more salt and water through urine than does the diuretic hydrochlorothiazide. Thus, furosemide has greater efficacy than hydrochlorothiazide.

A3: Potency—The power of a medicinal agent to produce the desired effects.

A4: Generics—Small (single molecule) or low molecular weight chemically synthesized compounds consisting of a simple, well defined structure that is independent of the manufacturing process and easy to characterize completely

A5: Immunogenicity—Immunogenicity is the ability of an agent, such as an epitope or an antigen, to illicit an immune response within the body. This can have both therapeutic and adverse consequences. Immunogenicity can also be categorized as the degree to which the foreign substance has elicited an immune response within the body and can be measured in things such as immunoassay drug testing.

A6: Interchangeability—In medicine, this refers to drugs that contain the same amount of the same active ingredients, possesse comparable pharmacokinetic properties, have the same clinically significant formulation characteristics, and are to be administered in the same way as the drug prescribed.

A7: Pharmacovigilance—Pharmacovigilance is defined as the science and activities relating to the detection, assessment, understanding and prevention of adverse effects or any other drug-related problem.

A8: Drug patent—When a pharmaceutical company first develops a new drug to be used for a disease condition, it is initially sold under a brand name by which the Clinicians can prescribe the drug for use by patients. The drug is covered under patent protection, which means that only the pharmaceutical company that holds the patent is allowed to manufacture, market the drug and eventually make profit from it.

## Appendix B. (Additional Tables)

**Table A1:** List of Biosimilars approved till 2018.

Name of Biosimilar Product	Approved By	Date of Approval	Manufacturer
**Omnitrope**	EMA	April 2006	Sandoz
**Binocrit**	EMA	August 2007	Sandoz
**Epoetin alfa Hexal**	EMA	August 2007	Hexal
**Abseamed**	EMA	August 2007	Medice
**Silapo**	EMA	December 2007	Stada
**Retacrit**	EMA	December 2007	Hospira UK
**Ratiograstim**	EMA	September 2008	Ratiopharm
**Tevagrastim**	EMA	September 2008	Teva
**Biograstim**	EMA	September 2008	CT Arzneimittel
**Filgrastim ratiopharm**	EMA	September 2008	Ratiopharm
**Filgrastim hexal**	EMA	February 2009	Hexal
**Zarzio**	EMA	February 2009	Sandoz
**Nivestim**	EMA	June 2010	Hospira UK
**Remsima**	EMA	September 2013	Celltrion
**Ovaleap**	EMA	September 2013	Teva
**Grastofil**	EMA	October 2013	Apobiologix
**Bemfola**	EMA	March 2014	Finox Biotech
**Accofil**	EMA	July 2014	Accord
**Abasaglar**	EMA	September 2014	Eli Lilly
**Zarxio**	US FDA	March 2015	Sandoz
**Basaglar**	US FDA	December 2015	Eli Lilly
**Benepali**	EMA	January 2016	Samsung Bioepis
**Inflectra**	US FDA	April 2016	Celltrion
**Flixabi**	EMA	May 2016	Samsung Bioepis
**Inhixa**	EMA	July 2016	Techdow Europe AB
**Thorinane**	EMA	July 2016	Pharmathen S.A
**Erelzi**	US FDA	August 2016	Sandoz
**Amjevita**	US FDA	September 2016	Amgen
**Movymia**	EMA	November 2016	Stada
**Terrosa**	EMA	November 2016	Gedeon Richter
**Amgevita**	EMA	January 2017	Amgen
**Solymbic**	EMA	March 2017	Amgen
**Renflexis**	US FDA	April 2017	Merck & Co. Inc.
**Cyltezo**	US FDA	April 2017	Boehringer Ingelheim
**Truxima**	EMA	August 2017	Celltrion
**Mvasi**	US FDA	September 2017	Genentech
**Ogivri**	US FDA	December 2017	Mylan
**Retacrit**	US FDA	May 2018	Pfizer Hospira
**Fulphila**	US FDA	June 2018	Mylan NV

**Table A2:** Current Challenges to Biosimilar Introduction and their Potential Solutions.

Challenges	Solutions
Reluctance of drug substitution	Promotion of Clinician and patient education of biosimilars, provision of sufficient clinical data to raise assurance and improved Clinician acceptance
Complex manufacturing process	Investment in advanced production techniques as well as outsourcing of biosimilar technology. Approval of related regulatory authority in even minor variations in the production process
Regulatory complications and lack of clarity	Establishment of proper communication with regulatory authorities and connected stakeholders. Allowance for open platforms of dialogue between all parties
Intellectual property rights	Design of strong unified and unitary patent litigation systems
Reach of biosimilar manufacturers to patients	Encouragement of manufacturers to differentiate their product from other service offerings and provide sufficient evidence of cost effectiveness, quality, safety, etc

**Table A3:** Current Landscape of Biosimilars in Treatment of Diseases.

Name of Biosimilar Product	Reference Product	Class of Biological Medicine	International Non-Proprietary Name (INN)	Indications
Zarxio	Neupogen	Granulocyte Colony Stimulating Factor (G-CSF)	Filgrastim	HIV or drug therapy induced neutropenia
Zarzio				Certain forms of cancer
				Severe chronic congenital neutropenia
				Hematopoietic stem cell transplantation
	Remicade	Tumor Necrosis Factor (TNF) alpha blocker	Infliximab	Psoriasis
				Rheumatoid arthritis
Inflectra				Psoriatic arthritis
Remsima				Crohn’s disease
				Ulcerative colitis
				Ankylosing spondilitis
Erelzi	Enbrel	Tumor Necrosis Factor (TNF) alpha blocker	Etanercept	Rheumatoid arthritis
				Polyarticular juvenile idiopathic
				arthritis
				Plaque psoriasis
				Ankylosing spondylitis Psoriatic arthritis
	Humira	Tumor Necrosis Factor (TNF) alpha blocker	Adalimumab	Rheumatoid arthritis
				Psoriatic arthritis
				Ankylosing spondylitis
Amjevita				Crohn’s disease
Amgevita				Ulcerative colitis
				Juvenile idiopathic arthritis
				Plaque psoriasis
				Uveitis
	Remicade	Tumor Necrosis Factor (TNF) alpha blocker	Infliximab	Psoriasis
				Rheumatoid arthritis
Renflexis				Ankylosing spondilitis
Flixabi				Psoriatic arthritis
				Crohn’s disease
				Ulcerative colitis
Cyltezo	Humira	Tumor Necrosis Factor (TNF) alpha blocker	Adalimumab	Rheumatoid arthritis
				Psoriatic arthritis
				Ankylosing spondylitis
				Crohn’s disease
				Ulcerative colitis
				Juvenile idiopathic arthritis
				Plaque psoriasis
				Uveitis
Mvasi	Avastin	Monoclonal Antibody (MAb)	Bevacizumab	Metastatic Colorectal cancer
				Metastatic HER2-negative Breast cancer
				Metastatic Renal cell carcinoma
				Second line treatment of Glioblastoma
				Second line treatment of Metastatic
				Colorectal cancer
				First line treatment of Non-Small Cell
				Lung Cancer (NSCLC)
Omnitrope	Genotropin	Growth Hormone (GH)	Somatotropin	Inadequate endogenous GH secretion
				Prader—Willi Syndrome
				Turner Syndrome Childhood/adult onset
				GH deficiency
				Pituitary dwarfism
Binocrit	Eprex	Erythropoietin (EPO)	Epoetin alpha	Anemia due to Chronic Kidney Disease (CKD)
				Anemia due to Zidovudine in patients with HIV infection
				Anemia due to chemotherapy in
				patients with cancer
				Reduction of allogenic red blood cell transfusions
Epoetin alfa Hexal	Eprex	Erythropoietin (EPO)	Epoetin alpha	Anemia due to Chronic Kidney Disease (CKD)
				Anemia due to Zidovudine in patients with HIV infection
				Anemia due to chemotherapy in patients with cancer
				Reduction of allogenic red blood cell transfusions
Abseamed	Eprex	Erythropoietin (EPO)	Epoetin alpha	Anemia due to Chronic Kidney Disease (CKD)
				Anemia due to Zidovudine in patients with HIV infection
				Anemia due to chemotherapy in patients with cancer
				Reduction of allogenic red blood cell transfusions
Silapo	Eprex	Erythropoietin (EPO)	Epoetin zeta	Anemia due to Chronic Kidney Disease (CKD)
				Anemia of renal origin accompanied by clinical symptoms in patients with renal insufficiency
				Reduction of transfusion requirements in patients receiving chemotherapy for tumours, malignant lymphoma or
				multiple myeloma
Retacrit	Eprex	Erythropoietin (EPO)	Epoetin zeta	Anemia due to Chronic Kidney Disease (CKD)
				Anemia of renal origin accompanied by clinical symptoms in patients with renal insufficiency
				Reduction of transfusion requirements in
				patients receiving chemotherapy for
				tumours, malignant lymphoma or multiple myeloma
Ratiograstim	Neupogen	Granulocyte Colony Stimulating Factor (G-CSF)	Filgrastim	HIV or drug therapy induced neutropenia
				Certain forms of cancer
				Severe chronic congenital neutropenia
				Hematopoietic stem cell transplantation
Tevagrastim	Neupogen	Granulocyte Colony Stimulating Factor (G-CSF)	Filgrastim	HIV or drug therapy induced neutropenia
				Certain forms of cancer
				Severe chronic congenital neutropenia
				Hematopoietic stem cell transplantation
Biograstim	Neupogen	Granulocyte Colony Stimulating Factor (G-CSF)	Filgrastim	HIV or drug therapy induced neutropenia
				Certain forms of cancer
				Severe chronic congenital neutropenia
				Hematopoietic stem cell transplantation
Filgrastim ratiopharm	Neupogen	Granulocyte Colony Stimulating Factor (G-CSF)	Filgrastim	HIV or drug therapy induced neutropenia
				Certain forms of cancer
				Severe chronic congenital neutropenia
				Hematopoietic stem cell transplantation
Filgrastim hexal	Neupogen	Granulocyte Colony Stimulating Factor (G-CSF)	Filgrastim	HIV or drug therapy induced neutropenia
				Certain forms of cancer
				Severe chronic congenital neutropenia
				Hematopoietic stem cell transplantation
Accofil	Neupogen	Granulocyte Colony Stimulating Factor (G-CSF)	Filgrastim	HIV or drug therapy induced neutropenia
				Certain forms of cancer
				Severe chronic congenital neutropenia
				Hematopoietic stem cell transplantation
Nivestim	Neupogen	Granulocyte Colony Stimulating Factor (G-CSF)	Filgrastim	HIV or drug therapy induced neutropenia
				Certain forms of cancer
				Severe chronic congenital neutropenia
				Hematopoietic stem cell transplantation
Ovaleap	Gonal-f	Follicle Stimulating Hormone (FSH)	Follitropin alpha	Polycystic ovary syndrome
Grastofil	Neupogen	Granulocyte Colony Stimulating Factor (G-CSF)	Filgrastim	HIV or drug therapy induced neutropenia
				Certain forms of cancer
				Severe chronic congenital neutropenia
				Hematopoietic stem cell transplantation
Bemfola	Gonal-f	Follicle Stimulating Hormone (FSH)	Follitropin alpha	Induction of ovulation in anovulatory
				infertile patient
				Induction of pregnancy in anovulatory infertile patient
Abasaglar Basaglar	Lantus	Human Insulin Hormone	Insulin glargine	Type 1 diabetes (in adults and children)
				Type 2 diabetes (in adults when basal
				insulin is needed)
Benepali	Enbrel	Tumor Necrosis Factor (TNF) alpha blocker	Etanercept	Rheumatoid arthritis
				Polyarticular juvenile idiopathic arthritis
				Plaque psoriasis
				Ankylosing spondylitis Psoriatic arthritis
Lusduna	Lantus	Human Insulin Hormone	Insulin glargine	Type 1 diabetes (in adults and children)
				Type 2 diabetes (in adults when basal
				insulin is needed)
Inhixa	Clexane	Low molecular weight heparin (Anticoagulant)	Enoxaparin Sodium	Prophylaxis of deep vein thrombosis (DVT)
				Prophylaxis of thromboembolic complications
Thorinane	Clexane	Low molecular weight heparin (Anticoagulant)	Enoxaparin Sodium	Prophylaxis of deep vein thrombosis (DVT)
				Prophylaxis of thromboembolic complications
Solymbic	Humira	Tumor Necrosis Factor (TNF) alpha blocker	Adalimumab	Rheumatoid arthritis
				Psoriatic arthritis
				Ankylosing spondylitis
				Crohn’s disease
				Ulcerative colitis
				Juvenile idiopathic arthritis
				Plaque psoriasis
				Uveitis
Truxima	MabThera	Monoclonal Antibody (MAb)	Rituximab	Chronic lymphocytic leukemia
				Rheumatoid arthritis
				Non-Hodgkin’s lymphoma
				Granulomatos with Polyangiitis (GPA) and microscopic Polyangiitis (MPA)
Movymia	Forsteo	Para-Thyroid Hormone (PTH)	Teriparatide	Post-menopausal osteoporosis
				Idiopathic osteoporosis in men
				Hypogonodal osteoporosis in men
				Glucocorticoid induced osteoporosis
Terrosa	Forsteo	Para-Thyroid Hormone (PTH)	Teriparatide	Post-menopausal osteoporosis
				Idiopathic osteoporosis in men Hypogonodal osteoporosis in men
				Glucocorticoid induced osteoporosis

**Table A4:** Differences between small molecule drugs and biological drugs.

Characteristics	Small Molecule Drugs	Large Molecular Drugs
Size	Single molecule, hence small	Combination of closely
	Low molecular weight	related molecules, hence large
		Large molecular weight
Structure	Simple, well defined, regardless of manufacturing process	Complex (heterogeneous), defined by exact manufacturing process
Modification	Well defined	Wider range of options
Stability	Stable	Unstable, sensitive to external conditions
Characterisation	Can be characterized completely	Cannot be characterised completely because of molecular composition and heterogeneity
Immunogenicity	Usually non-immunogenic	Immunogenic
Manufacturing	Produced by chemical synthesis	Produced in living cell culture
	Foreseeable chemical process	Difficult to control process from starting material to final API
	Duplicate copy can be made	Impossible to ensure duplicate copy
Susceptibility to contamination during manufacture	Low	High
Sensitivity to physical factors (heat, light)	Low	High
Clinical behaviour	Well defined mode of action	Complex modes of action
Species	Interdependent	Specific
Absorption	More rapid	Slower
Distribution	High	Limited
Metabolism	Metabolized to active and non-active metabolites	Broken down to endogenous amino acids
Disposition	Rarely target mediated	Mostly target mediated
Half-life	Shorter	Longer

**Table A5:** Differences between small molecule, generic, biological and biosimilar drugs.

	Pharmaceutical Industry		Biopharmaceutical Industry	Biosimilar Industry
Parameter	Small Molecule Drug	Generic Drug	Biological Drug	Biosimilar Drug
Synthesis	Production of	Copy from the	Manufactured in a living system, usually through recombinant DNA technology	Development derived from
	original chemical	original chemical		the original biological
	formula	formula		molecule
Size	100–1000 Da	100–1000 Da	10,000–300,000 Da	10,000–300,000 Da
Glycosylation process	Zero	Zero	Several	Several
Molecular structure	Simple	Simple	Complex	Complex
Ability to generate immunity	Low	Low	Medium–High	Medium–High
Drug development time	7–10 years	1–3 years	10–15 years	6–9 years
Characterisation via analysis in laboratory	N/A	There are techniques to identify similarity to the original drug	N/A	No identification technique for equality of the molecule
				Clinical studies
				are needed
