# Supplementary material for: An Empirical Analysis of the Perceived Challenges and Benefits of Introducing Biosimilars in Bangladesh: A Paradigm Shift
Source: Biomolecules. 2018 Sep 5;8(3):89. doi: 10.3390/biom8030089 (PMC6163433; doi:10.3390/biom8030089)
Supplement: Supplementary file 1 [file biomolecules-08-00089-s001.pdf]

BRAC UNIVERSITY
Inspiring Excellence

Biosimilar Perception, Challenge and Benefit Assessment
Questionnaire for Academicians
Department of Pharmacy
BRAC University

This survey aims to evaluate the perception, knowledge and awareness of biosimilar drugs among academicians from both public and private universities within Bangladesh. It seeks to determine the degree of inclusion of biosimilar education within the university curriculum. Your support and participation will be highly beneficial in our assessment of the challenges and benefits of biosimilar introduction in our country. We will appreciate your participation in this regard.

This Questionnaire will only require 5-7 minutes of your time.

This survey is being conducted solely for the purpose of research under the supervision of Dr. Eva Rahman Kabir, Professor and Chairperson, Department of Pharmacy, BRAC University and Mohammad Kawsar Sharif Siam, Senior Lecturer, Department of Pharmacy, BRAC University.

Statement of Confidentiality: Your participation in this research is confidential. The survey does not ask for any information that would identify you. In the event of any publication or presentation resulting from the research, no personally identifiable information will be shared because your name is in no way linked to your responses. This study has been approved by the Department of Pharmacy, BRAC University.

Voluntary Participation: Your decision to be in this research is voluntary. You can stop at any time. You do not have to answer any questions you do not want to answer.

Thank you.
Shannon Sherwin Moreino
ID: 12146040
Department of Pharmacy
BRAC University

Summary: Biosimilars are less costly imitations of large molecule drugs known as biologics, which are used to treat a range of diseases including cancer, rheumatoid arthritis, diabetes, and anemia. But they are different from generics in that they are not exact copies. Biosimilars are a relatively recent development in the pharmaceutical industry, but they are poised to be at the forefront of pharma technology. Countries such as Bangladesh with limited health budget, strong drug policies and a lower income earning population strongly seek to benefit from the rapid growth of these products within the local industry.
Survey Period: 4 months

Department of Pharmacy
Dhaka-1212
BRAC University

BRAC UNIVERSITY

Inspiring Excellence

Respondent's Name:

Designation: **Organization:**

1. **Choose one definition that reflects your understanding of biosimilars: [Tick any one answer]**

   a) A biologic that demonstrates equivalence with the original biodrug and has all the preclinical and clinical trials equal to those already performed with the original biodrug
   b) A biologic that demonstrates bioequivalence with an original biodrug and does not need clinical trials to be commercialized

2. **Among the following options, which do you feel are the major challenges associated with the introduction of biosimilars in the drug industry? [Multiple answers can be ticked]**

   a) Need for updated regulatory guidelines
   b) Need for improved pharmacovigilance systems to ensure drug safety
   c) Extrapolation of clinical data may be insufficient to determine effectiveness of biosimilar
   d) Complications during substitution of drug in patient therapy
   e) Economic and societal consequences of biosimilar use

3. **In your opinion, can a biosimilar and its reference biologic drug be used interchangeably? [Tick any one answer]**

   a) Yes
   b) No
   c) Not known

If you have selected "No", give a reason as to why you feel so:

Department of Pharmacy, BRAC

BRAC UNIVERSITY

Inspiring Excellence

4. **Among the following options, which do you feel are the advantages presented by the introduction of biosimilars in Bangladesh? [Multiple answers can be ticked]**

a) Lower cost
b) Commercialization approved with initial indication including all diseases previously approved for the innovator biodrug
c) Administration route different from that of the original biodrug
d) Lower therapeutic dose

5. **Is there course material on biologics and/or biosimilars included in the curriculum? If yes, what are the courses being taught that include information on biologics and/or biosimilars?**

Answer:

	Strongly Disagree	Disagree	Undecided	Agree	Strongly Agree
**If no, how much do you agree that they should be included? [Tick any one answer on Likert scale]**	1	2	3	4	5

Department of Pharmacy, BRACU

BRAC UNIVERSITY
Inspiring Excellence

6. In your opinion, how essential are comparability studies in the role of determining the effectiveness of biosimilar products? Are they sufficient to determine the safety and efficacy of the product?

Answer:

---Thank You---

Department of Pharmacy, BRACU

Inspiring Excellence

Biosimilar Perception, Challenge and Benefit Assessment
Questionnaire for Clinicians
Department of Pharmacy
BRAC University

This survey aims to evaluate the perception, knowledge and awareness of biosimilar drugs among clinicians currently practicing in hospitals within Bangladesh. It seeks to determine the feasibility of biosimilar introduction and prescription within the country. Your support and participation will be highly beneficial in our assessment of the challenges and benefits of biosimilar introduction in our country. We will appreciate your participation in this regard. Thank you.

This Questionnaire will only require 5-7 minutes of your time.

This survey is being conducted solely for the purpose of research under the supervision of Dr. Eva Rahman Kabir, Professor and Chairperson, Department of Pharmacy, BRAC University and Mohammad Kawsar Sharif Siam, Senior Lecturer, Department of Pharmacy, BRAC University.

Statement of Confidentiality: Your participation in this research is confidential. The survey does not ask for any information that would identify you. In the event of any publication or presentation resulting from the research, no personally identifiable information will be shared because your name is in no way linked to your responses. Furthermore, no prescription related information will be released. This study has been approved by the Department of Pharmacy, BRAC University.

Voluntary Participation: Your decision to be in this research is voluntary. You can stop at any time. You do not have to answer any questions you do not want to answer.

Thank you.
Shannon Sherwin Moreino
ID: 12146040
Department of Pharmacy
BRAC University

Summary: Biosimilars are less costly imitations of large molecule drugs known as biologics, which are used to treat a range of diseases including cancer, rheumatoid arthritis, diabetes, and anemia. But they are different from generics in that they are not exact copies. Biosimilars are a relatively recent development in the pharmaceutical industry, but they are poised to be at the forefront of pharma technology. Countries such as Bangladesh with limited health budget, strong drug policies and a lower income earning population strongly seek to benefit from the rapid growth of these products within the local industry.

Survey Period: 4 months

Department of Pharmacy
BRAC University

BRAC UNIVERSITY

Inspiring Excellence

Respondent's Name:	
Designation:	Organization:

1. Choose one definition that reflects your understanding of biosimilars: [Tick any one answer]

a) A biologic that demonstrates equivalence with the original biodrug and has all the preclinical and clinical trials equal to those already performed with the original biodrug
b) A biologic that demonstrates bioequivalence with an original biodrug and does not need clinical trials to be commercialized

2. Among the following options, which is the main source from which you have been made aware of the existence, applications and benefits of biosimilars [Tick any one answer]

a) Peer reviewed journal articles
b) Patient groups
c) Biotech companies
d) Medical conferences
e) Key Opinion Leaders (KOLs)
f) Medical associations
g) Popular press
h) Insurance companies
i) Others

3. Among the following options, which do you feel are the major challenges associated with the introduction of biosimilars in the drug industry? [Multiple answers can be ticked]

a) Need for updated regulatory guidelines
b) Need for improved pharmacovigilance systems to ensure drug safety
c) Extrapolation of clinical data may be insufficient to determine effectiveness of biosimilar
d) Complications during substitution of drug in patient therapy
e) Economic and societal consequences of biosimilar use

Department of Pharmacy, BRAC U

BRAC UNIVERSITY

Inspiring Excellence

4. In your opinion, can a biosimilar and its reference biologic drug be used interchangeably? [Tick any one answer]

a) Yes
b) No
c) Not known

If you have selected "No", give a reason as to why you feel so:

5. Among the following options, which do you feel are the advantages presented by the introduction of biosimilars in Bangladesh? [Multiple answers can be ticked]

a) Lower cost
b) Commercialization approved with initial indication including all diseases previously approved for the innovator biodrug
c) Administration route different from that of the original biodrug
d) Lower therapeutic dose

6. In your opinion, what would be the main reason for you to prescribe biosimilars? [Tick any one answer]

a) Trust towards manufacturers of biosimilars
b) Same safety profile as in original biologics
c) Same efficacy in original biologics
d) Cost advantage
e) Propitiousness of reimbursement authorities
f) Propitiousness of patient advocacy groups
g) Others

---Thank You---

Department of Pharmacy, BRACU

BRAC UNIVERSITY

Inspiring Excellence

Biosimilar Perception, Challenge and Benefit Assessment
Questionnaire for Industry Experts
Department of Pharmacy
BRAC University

This survey aims to evaluate the perception, knowledge and awareness of biosimilar drugs among industry experts from leading pharmaceutical companies within Bangladesh. It seeks to determine the feasibility of biosimilar introduction and manufacture within the country. Your support and participation will be highly beneficial in our assessment of the challenges and benefits of biosimilar introduction in our country. We will appreciate your participation in this regard. Thank you.

This Questionnaire will only require 5-7 minutes of your time.

This survey is being conducted solely for the purpose of research under the supervision of Dr. Eva Rahman Kabir, Professor and Chairperson, Department of Pharmacy, BRAC University and Mohammad Kawsar Sharif Siam, Senior Lecturer, Department of Pharmacy, BRAC University.

Statement of Confidentiality: Your participation in this research is confidential. The survey does not ask for any information that would identify you. In the event of any publication or presentation resulting from the research, no personally identifiable information will be shared because your name is in no way linked to your responses. Furthermore, no industry related information will be released. This study has been approved by the Department of Pharmacy, BRAC University.

Voluntary Participation: Your decision to be in this research is voluntary. You can stop at any time. You do not have to answer any questions you do not want to answer.

Thank you.
Shannon Sherwin Moreino
ID: 12146040
Department of Pharmacy
BRAC University

Summary: Biosimilars are less costly imitations of large molecule drugs known as biologics, which are used to treat a range of diseases including cancer, rheumatoid arthritis, diabetes, and anemia. But they are different from generics in that they are not exact copies. Biosimilars are a relatively recent development in the pharmaceutical industry, but they are poised to be at the forefront of pharma technology. Countries such as Bangladesh with limited health budget, strong drug policies and a lower income earning population strongly seek to benefit from the rapid growth of these products within the local industry.
Survey Period: 4 months

Department of Pharmacy
BRAC University

BRAC UNIVERSITY

Inspiring Excellence

**Respondent's Name:**	
**Designation:**	**Organization:**

1. Choose one definition that reflects your understanding of biosimilars: [Tick any one answer]

a) A biologic that demonstrates equivalence with the original biodrug and has all the preclinical and clinical trials equal to those already performed with the original biodrug
b) A biologic that demonstrates bioequivalence with an original biodrug and does not need clinical trials to be commercialized

2. Are Non – Comparable Biologics (NCBs) synonymous to biosimilars? [Tick any one answer]

a) Yes
b) No
c) Unaware

3. Among the following options, which do you feel are the major challenges associated with the introduction of biosimilars in the drug industry? [Multiple answers can be ticked]

a) Need for updated regulatory guidelines
b) Need for improved pharmacovigilance systems to ensure drug safety
c) Extrapolation of clinical data may be insufficient to determine effectiveness of biosimilar
d) Complications during substitution of drug in patient therapy
e) Economic and societal consequences of biosimilar use

4. Among the following options, which drivers do you feel would motivate clinicians to prescribe biosimilars? [Multiple answers can be ticked]

a) Lower price of the biosimilar in comparison with the innovator biodrug
b) Country of origin where the biosimilar has been manufactured
c) Certified approval for the biosimilar by the relevant authorities
d) Good manufacturing practices and high reputation of the manufacturer
e) Bioefficacy of the biosimilar drug
f) Safety of the biosimilar drug

BRAC UNIVERSITY

Inspiring Excellence

5. **In your opinion, can a biosimilar and its reference biologic drug be used interchangeably? [Tick any one answer]**

a) Yes
b) No
c) Not known

If you have selected "No", give a reason as to why you feel so:

6. **Among the following options, which practice do you feel biosimilar manufacturers can best employ to provide evidence to clinicians regarding the reliability of their product (especially in terms of non-immunogenicity)? [Tick any one answer]**

a) Provision of bioequivalent safety and efficacy data
b) Provision of data of Phase III clinical trial outcomes within a sample of the local population
c) Provision of evidence of strong GMP maintenance
d) Provision of evidence that WHO guidelines have been followed during product registration

7. **Among the following options, which patient education strategies do you feel would be the most effective in increasing patient awareness of the use of biosimilar drug products? [Multiple answers can be ticked]**

a) Public counselling campaigns through various appropriate media
b) Patient counselling programs sponsored by pharmaceutical companies and hospitals
c) Patient education provided by hospital personnel during drug prescription
d) Patient education through open seminars or symposiums held by academicians from universities citywide

BRAC UNIVERSITY

Inspiring Excellence

8. Among the following options, which do you feel are the advantages presented by the introduction of biosimilars in Bangladesh? [Multiple answers can be ticked]

a) Lower cost
b) Commercialization approved with initial indication including all diseases previously approved for the innovator biodrug
c) Administration route different from that of the original biodrug
d) Lower therapeutic dose

Answer the following questions by ticking any one answer from the provided Likert scale

	**Strongly Disagree**	**Disagree**	**Undecided**	**Agree**	**Strongly Agree**
**9. How much do you agree that the introduction of biosimilars is feasible in our country?**	1	2	3	4	5
**10. In your opinion, how much would you agree that Bangladesh has these regulatory requirements clearly outlined?**	1	2	3	4	5

--- Thank You ---
